# Lemierre-like syndrome after soft tissue infection due to methicillin-resistant *Staphylococcus aureus*: A case report and literature review

**DOI:** 10.1097/MD.0000000000037006

**Published:** 2024-02-16

**Authors:** Xosse Carreras, Andrea S. Salcedo, Linda Ponce-Rosas, Jose A. Gonzales-Zamora, Nelson Diaz, Jorge Alave

**Affiliations:** a School of Medicine, Universidad Peruana de Ciencias Aplicadas, Lima, Peru; b Department of Medicine, Hamilton Medical Center, Dalton, GA 30720; c Peruvian American Medical Society, Albuquerque, NM; d Division of Infectious Diseases, Department of Medicine, University of Miami, Miller School of Medicine, FL 33136; e School of Medicine, Universidad Peruana Union, Lima, Peru; f Department of Internal Medicine, Clínica Good Hope, Lima, Peru.

**Keywords:** case report, Lemierre’s syndrome, *methicillin-resistant Staphylococcus aureus*, septic emboli

## Abstract

**Rationale:**

Lemierre-like syndrome (LLS) is characterized by bacteremia, septic thrombophlebitis of the internal jugular vein, and metastatic abscesses. In contrast to classic Lemierre syndrome, sources of infection are not related to oropharyngeal infections, as are frequent soft tissue infections. In recent years, *Staphylococcus aureus* has been identified as an emergent pathogen that causes this syndrome. The mortality rate of LLS caused by this pathogen is approximately 16%. Timely diagnosis, antibiotic treatment, and infection control are the cornerstones to treat LLS. Anticoagulant therapy as adjuvant treatment remains controversial.

**Patient concerns:**

A 31-year-old woman from California, United States (US), was admitted to the emergency room with a history of 2 days of fever and severe throbbing pain in the left cervical region. Thorax and neck CT tomography revealed confluent cavities suggestive of septic embolism in the lungs and a filiform thrombus in the lumen of the left internal jugular vein, with moderate swelling of the soft and muscular tissues. Methicillin-resistant *Staphylococcus aureus* (MRSA) was isolated from the blood culture.

**Diagnosis:**

The thrombus in the internal jugular vein associated with cellulitis in the neck and multiple cavitary lesions in the lungs support the diagnosis of LLS caused by MRSA with septic embolization.

**Interventions:**

During treatment, the patient received vancomycin IV for 25 days and returned to the US with linezolid orally. In addition, assisted video-thoracoscopy and bilateral mini-thoracotomy with pleural decortication were performed for infectious source control, where 1700cc of purulent pleural fluid was drained.

**Outcomes:**

The patient was discharged with optimal evolution.

**Lessons:**

LLS should be suspected in patients with skin and soft tissue infections who develop thrombosis or metastatic infections. MRSA infections should be considered in patients from areas where this pathogen is prevalent.

## 1. Introduction

Lemierre’s syndrome (LS) is an acute oropharyngeal infection characterized by bacteremia, septic thrombophlebitis of the internal jugular vein (IJV), and metastatic abscesses that mainly affect the lungs.^[1]^ The most common etiologic agent is *Fusobacterium necrophorum* with a prevalence of up to 30%.^[2]^ Other isolated organisms include *Bacteroides and Streptococcus*.^[2]^

In recent years, similar cases unrelated to oropharyngeal infections, referred to as Lemierre-like syndrome (LLS), have been increasingly reported.^[1]^ The etiology of LLS can be variable, highlighting *Staphylococcus aureus* as an emerging pathogen with a prevalence of 5%.^[3]^ Methicillin-resistant *Staphylococcus aureus* (MRSA) has been reported in 2% of cases.^[4,5]^ It is important to recognize the high mortality rate associated with this condition, which has been reported to be as high as 16%.^[1,6]^

The current treatment for LLS is based on empirical antibiotic therapy directed toward the most frequent pathogens. Management of IJV thrombosis is controversial, with some experts recommending anticoagulation as adjuvant treatment.^[7]^ Here, we present a rare case of LLS in an immunocompetent young woman secondary to a soft tissue infection of the neck, for whom antibiotic treatment was timely. LLS should be suspected in patients with skin and soft tissue infections who develop thrombosis and metastatic disease.

## 2. Case report

A 31-year-old woman from California, United States, was admitted to the emergency room with a history of 2 days of fever and severe throbbing pain in the left cervical region, associated with paresthesia in the ipsilateral upper extremity. She denied any preexisting diseases or previous hospitalization. In the last 6 months, she traveled to Costa Rica, Colombia, San Francisco, Cusco, and Lima. She reported social use of lysergic acid, diethylamide, ecstasy, and poppers 2 weeks before admission, but denied intravenous drug use.

**Figure 1.**
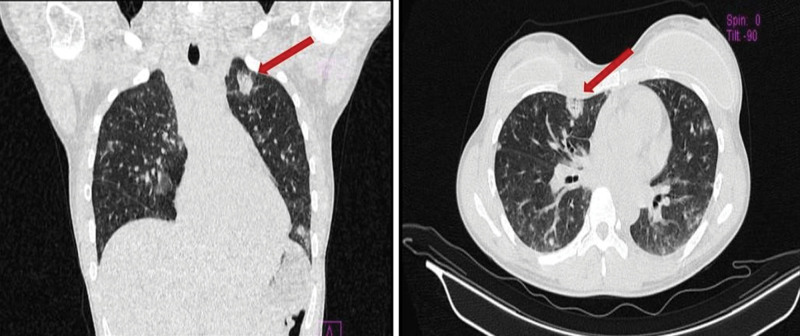
A non-contrast thoracic CT scan showed multiple bilateral nodules (red arrows) on admission.

**Figure 2.**
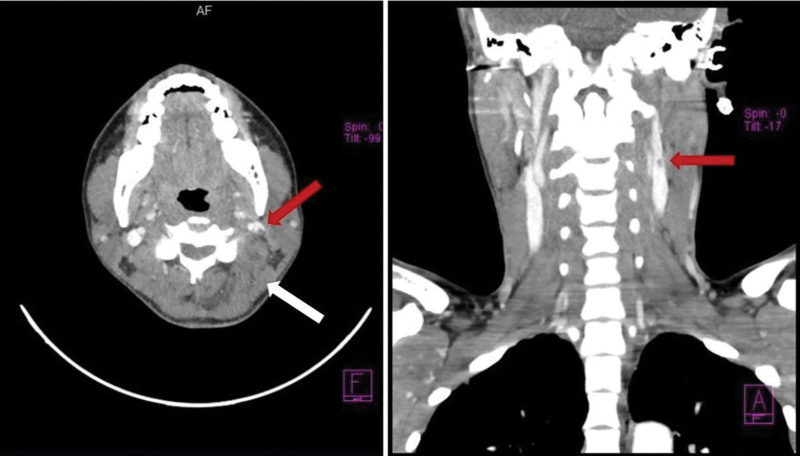
On admission, a contrast neck CT scan showed increased volume and inflammation of subcutaneous cellular tissue (white arrow) and hypodensity in the jugular vein (red arrows).

On initial physical examination, the patient presented with a fever of 39°C. The remaining vital signs were within normal ranges. She was oriented toward the place, time, and person. In the left cervical region, swelling, erythema, and warmth are associated with severe tenderness upon palpation. No masses were noted. The lung examination results were normal. On the 2nd day of hospitalization, she presented with bilateral pleuritic pain and mild dyspnea. On examination, decreased breath sounds with dullness on percussion on both bases were observed. Oxygen saturation dropped to 93%, and oxygen support was initiated with a nasal cannula at 2 L/min. Laboratory studies showed marked leukocytosis with left shift (25.68 × 10^3^ cell/µL) and neutrophilia (23.63 × 10^3^ cell/µL). C-reactive protein (54.58 mg/L) and procalcitonin (5.4 ng/mL) levels were elevated, and prothrombin (17.5 s) and activated partial thromboplastin (51.5 s) levels were prolonged. HIV ELISA and urine toxicology tests were negative. Non-contrast CT of the thorax revealed bilateral diffuse nodules (Fig. 1). Contrast-enhanced CT scan of the neck revealed inflammation of the soft tissue associated with hypodensity in the IJV (Fig. 2). At this point, empiric antibiotic treatment was initiated with vancomycin (1 g every 12 h) and piperacillin-tazobactam (PIP-TZB) 4.5 g every 6 hours due to suspected soft tissue infection. Blood culture obtained on admission was positive on day 3 of hospitalization for methicillin-resistant *Staphylococcus aureus* (MRSA) sensitive to vancomycin (MIC less than 0.5 µg/mL). Consequently, PIP-TZB was discontinued.

**Figure 3.**
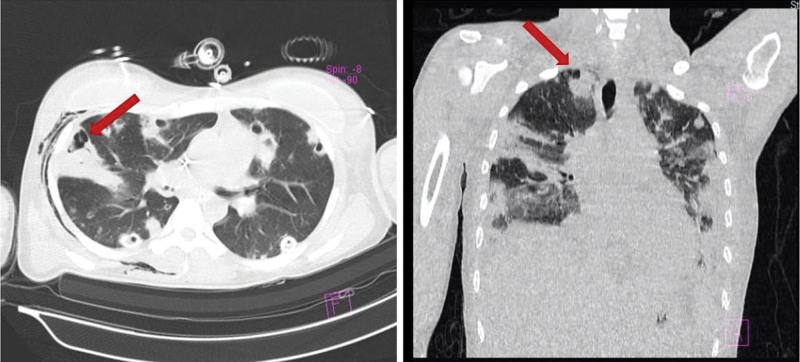
On day 5th of hospitalization, a contrast thoracic CT scan showed multiple necrotic areas with confluent cavities in both lungs (red arrows) and massive pleural effusion.

**Figure 4.**
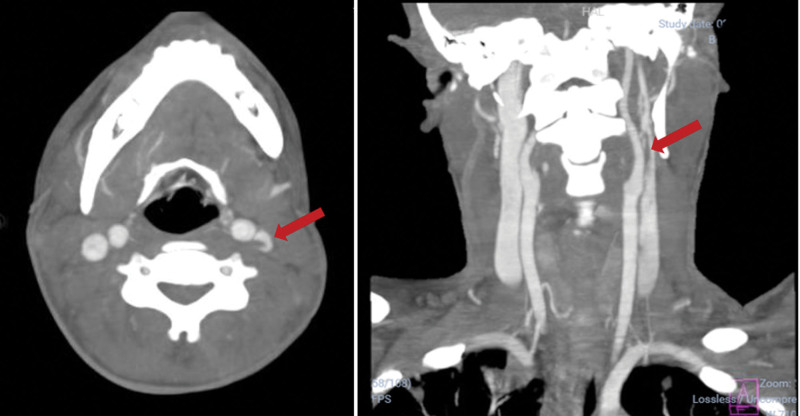
On day 5th of hospitalization, a neck angiography showed a hypodensity in the lumen of the left internal jugular vein, which was consistent with a filiform thrombus (red arrows).

Due to the development of intense bilateral pleuritic pain, oxygen desaturation, and persistent neck pain, a thoracic and neck CT scan was performed, which did not reveal pulmonary thromboembolism but showed bilateral multiform inflammatory processes with areas of necrosis and secondary confluent cavities suggestive of septic embolism and extensive bilateral pleural effusion (Fig. 3). In addition, it revealed a filiform thrombus in the lumen of the left internal jugular vein and moderate swelling of soft and muscular tissues (Fig. 4). These findings led to the diagnosis of LLS caused by MRSA with septic embolization. Given the massive pleural effusion and the high suspicion of empyema, a bilateral mini-thoracotomy with pleural decortication was performed by assisted video-thoracoscopy, where 1700cc of purulent pleural fluid was drained and cultured. Pleural fluid analysis showed an exudate with a predominance of polymorphonuclear cells (85%, 9950 mm^3^) and an LDH level of 989 µ/L. Gram and acid-fast stains were negative. Pleural fluid culture showed no growth of common bacterial pathogens, tuberculosis, or fungi. GeneXpert results were also negative. The pleural fluid ADA level was 17.6 IU/L, which is within the normal range (36–229.7 IU/L). The patient had positive blood cultures for 8 days and finally cleared bacteremia after surgical drainage. After surgery, the patient was admitted to the intensive care unit where she was placed on mechanical ventilation. During the following days, the respiratory parameters improved, and she was extubated on day 8. Ten days later, the patient was transferred to an internal medicine ward.

Owing to the development of septic thrombophlebitis, the team decided to treat the patient with antibiotics for 28 days. During hospitalization, intravenous vancomycin was administered for 25 days. On discharge, vancomycin was switched to oral linezolid (600 mg every 12 hours). She returned to California. At the monthly follow-up, the patient reported sustainable clinical improvement and no medication-related side effects.

## 3. Discussion

LLS caused by MRSA has emerged as a disease over the last several years. It usually affects young adults aged 16 to 25 years, equally affecting both male and female patients. Community-acquired MRSA is more virulent and aggressive than healthcare-acquired MRSA, with more thrombotic complications.^[8]^

Van Hoecke et al^[1]^ described the clinical features and outcomes of 24 patients with LLS caused by *S. aureus* and found that more than half of the cases presented oropharyngeal infection as the source, and the rest were sinusitis, cellulitis, furunculosis, infected hematoma in the sternocleidomastoid muscle, and neck abscesses, as reported by Gore.^[5]^ In our patient, the LLS originated from an episode of neck cellulitis. This should be suspected in cases of neck pain or marked swelling and tenderness from the jaw to the sternocleidomastoid muscle in patients with severe sepsis. The diagnosis is mostly confirmed by imaging, as in the present case.^[7]^ A significant LLS complication is metastatic infection of the bones, joints, and meninges, mainly to the lungs (97%),^[5]^ leading to pulmonary abscess or empyema.^[1]^

Given the indolent LLS course and obscurity of symptoms, a high index of clinical suspicion is required.^[9]^ The gold standard for diagnosis is identification of microorganisms by blood culture.^[10]^ However, imaging findings such as IJV thrombosis and/or septic pulmonary emboli can precede the blood culture results, as in our case.^[9]^ Although neck CT with contrast is the imaging modality of choice for IJV thrombosis detection, ultrasonography can also be useful for the assessment of thrombus extension.^[9]^

Neck cellulitis is a rare primary cause of LLS. A PubMed search with the terms (“Lemierre” and “neck cellulitis” or “soft tissue”) yielded 13 case reports (Table 1). Eleven patients had secondary soft tissue infection,^1,10,11,13,14,16–19]^ while the other 2 cases were secondary to osteomyelitis^[12]^ and oral ulcers.^[15]^ Their ages varied between 5 and 74 years. Almost half of the patients were male. In the cases described, the time of illness was between several hours and a month; however, most patients pursued medical attention in the 1st week. The most common symptom described was fever, including in our case, followed by neck pain and swelling. Furthermore, only 1 patient had known immunosuppression.^[11]^ The primary infection varied widely, including 5 cases of neck abscess^[13,14,18]^ and 3 cases of neck cellulitis,^[11,16]^ as in our case. *Staphylococcus aureus* was the most reported pathogen, including 6 cases of MRSA,^[10,14,15,17,19]^ 2 MSSA,^[1,13]^ and 1 *S. aureus* with an unknown resistance profile,^[18]^ followed by *Klebsiella pneumoniae* in 2 cases.^[11,18]^ All the patients received different combinations of antimicrobial therapies. In this review, we found 4 cases of anticoagulation therapy.^[13,16–18]^ Despite the high historical mortality reported for this syndrome, we found that almost all patients recovered.

**Table 1:** Salient features of 14 patients with Lemierre-like syndrome after soft tissue infection

References	Country	Age	Sex	Duration of illness	Clinical features	Immune status	Primary infection	Pathogen (source)	Antimicrobial therapy	Anticoagulation	Outcome
Singaporewalla et al^[11]^	Singapore	68	M	1 week	Painfull swelling of the neck and fever.	Uncontrolled diabetes	Neck cellulitis	*Klebsiella pneumoniae* (blood, abscess)	Ceftriaxone, Metronidazole, Amoxicillin/Clavulinic acid	No	Recovered
Nakamichi et al^[12]^	Japan	71	F	1 month	Retroauricular pain, fever.	Immunocompetent	Cervical osteomyelitis	None	Panipenem/betamoprin, Clindamycin, Linezolid, Meropenem, Ampicilin/Sulbactam	No	Recovered
Root et al^[13]^	USA	10 months	F	5 days	Intermittent fever, upper respiratory illness symptoms, decreased oral intake and refusal to move her head to the right.	Immunocompetent	Neck abscess	MSSA (blood)	Vancomycin, Cefuroxime, Clindamycin, Nafcillin	Heparin	Recovered
Jariwala et al^[14]^	USA	13 months	F	6 days	Fever, chills, swollen neck, and nonproductive cough.	Immunocompetent	Neck abscess	MRSA (abscess)	Ceftriaxone, Amoxicilin/clavulante, Vancomycin, Clindamycin, Piperacilin/Tazobactam	No	Recovered
Abhishek et al^[10]^	Brazil	24	M	3 days	Painful swelling of the right neck and shoulder area, fever, nausea, and lethargy.	Inmunocompetent	Infected neck hematoma	MRSA (blood, wound, sputum)	Vancomycin, Piperacilin/Tazobactam, Clindamycin	Heparin	Recovered
Herek et al^[15]^	USA	33	F	Several hours	Marked face and neck swelling, a perioral rash, dyspnea, and hoarseness.	Inmunocompetent	Oral ulcers	MRSA (blood, wound)	Piperacilin/Tazobactam, Vancomycin	No	Recovered
Risoud et al^[16]^	France	34	F	Several hours	Swelling of the left cheek and neck, fever, and trismus.	Inmunocompetent	Head and neck cellulitis/ Tonsilitis	Streptococcus constellatus (blood)	Ceftriaxone, Metronidazole, Amoxicilin/Clavulanate	Heparin, Vitamin K antagonits	Recovered
Al-Hebshi et al^[17]^	Pakistan	5 months	M	2 days	Fever, poor oral intake, and swelling of the neck.	Inmunocompetent	Retropharyngeal and para-pharyngeal abscess	MRSA (abscess)	Cefazolin, Vancomycin, Meropenem, Linezolid	Enoxaparin	Recovered
Kim et al^[18]^	South Korea	58	M	Unknown	Fever, neck swelling, and tenderness	Unknown	Neck abscess	None	None	None	Lost to follow-up
Kim et al^[18]^	South Korea	74	M	Unknown	Facial swelling	Unknown	Neck abscess	*Staphylococcus aureus*, Gram (+) cocci, Gram (–) cocci (Blood).	IV antibiotics (not described)	None	Recovered
Kim et al^[18]^	South Korea	76	F	Unknown	Fever, neck Swelling, and Tenderness.	Unknown	Neck abscess	*Klebsiella pneumoniae* (blood)	Intravenous antibiotics (not described)	Anticoaguation	Recovered
Van Hoecke et al^[1]^	Belgium	70	M	4 days	Neck pain, fever.	Inmunocompetent	Sternocleidomastoid muscle myositis	MSSA (blood)	Amoxicilin/clavulanic acid,, Flucloxacilin	None	Recovered
Kizhner et al^[19]^	USA	16	M	5 days	Neck pain, dry cough, and generalized malaise.	Inmunocompetent	Muscle injury	MRSA (blood)	Ceftriaxone, Azithromycin, Vancomycin, Rifampin	None	Recovered
Present case	Peru	32	F	2 days	Fever, painfull neck, paresthesia in upper extremity	Inmunocompetent	Neck cellulitis	MRSA (blood)	Vancomycin, Piperacilim/Tazobactam, Linezolid	None	Recovered

MRSA: methicillin-resistant *S. aureus*

Treatment is based on empirical broad-spectrum antibiotic therapy, which should be initiated as soon as possible.^[10]^ Empirical antibiotics should include both anaerobic and gram-positive bacteria. Subsequently, treatment can be tailored depending on the susceptibility profile of the isolated microorganisms.^[10]^ Several antibiotics can be used to treat MRSA, including vancomycin, linezolid, telavancin, and daptomycin; however, the latter should not be used when septic pulmonary thrombophlebitis is detected. In this case, vancomycin, followed by linezolid, was administered for 4 weeks. Antibiotic treatment should be continued until clot resolution, which usually occurs within 4 to 6 weeks.^[10,20]^ In addition to antibiotic therapy, it is crucial to achieve source of infection control to eradicate infection. In their case series, Van Hoecke et al^[1]^ reported that 13 of 25 patients (52%) underwent surgical intervention to some degree. In our case, pleural drainage was 1 of the most important components of treatment, which led to a complete microbiological and clinical cure.

Anticoagulation therapy is controversial because venous thrombosis is secondary to endothelial dysfunction caused by local infection by inflammatory factors.^[5,9]^ Most patients reported in the literature were administered anticoagulation; however, the mortality in the non-anticoagulation group was lower than that in the patients who received anticoagulation therapy (11% vs 19%).^[1,9]^ Given the lack of strong evidence in favor of anticoagulation, we decided not to initiate this therapy, which did not have any negative effect on the patient’s recovery.

## 4. Conclusion

It is necessary to increase awareness of LLS, which should be suspected in patients with skin and soft tissue infections who develop thrombosis and metastatic infections. Likewise, MRSA infection should be considered as a possible etiologic agent, especially in patients from areas where MRSA is prevalent in the community. Therefore, antibiotic therapy against MRSA is warranted. Anticoagulation remains controversial, and further studies are needed to evaluate its effectiveness as an adjunctive therapy in LLS.

## Author contributions

**Conceptualization:** Xosse Carreras, Andrea S. Salcedo.

**Investigation:** Xosse Carreras, Andrea S. Salcedo, Linda Ponce-Rosas, Jose A. Gonzales-Zamora, Nelson Diaz, Jorge Alave.

**Writing—original draft:** Xosse Carreras, Andrea S. Salcedo, Linda Ponce-Rosas, Jose A. Gonzales-Zamora, Nelson Diaz, Jorge Alave.

**Writing—review & editing:** Xosse Carreras, Andrea S. Salcedo, Linda Ponce-Rosas, Jose A. Gonzales-Zamora, Nelson Diaz, Jorge Alave.

**Supervision:** Jose A. Gonzales-Zamora, Nelson Diaz, Jorge Alave.

## References

[1] Van Hoecke F Lamont B Van Leemput A. A Lemierre-like syndrome caused by Staphylococcus aureus: an emerging disease. Infect Dis (Lond). 2020;52:143–51. 31749395 10.1080/23744235.2019.1691255

[2] Bentley TP Brennan DF. Lemierre’s syndrome: methicillin-resistant Staphylococcus aureus (MRSA) finds a new home. J Emerg Med. 2009;37:131–4. 18280087 10.1016/j.jemermed.2007.07.066

[3] Correia MS Sadler C. Methicillin-resistant staphylococcus aureus septic internal jugular thrombophlebitis: updates in the etiology and treatment of lemierre’s syndrome. J Emerg Med. 2019;56:709–12. 31229258 10.1016/j.jemermed.2019.03.031

[4] Johannesen KM Bodtger U. Lemierre’s syndrome: current perspectives on diagnosis and management. Infect Drug Resist. 2016;9:221–7. 27695351 10.2147/IDR.S95050 PMC5028102

[5] Gore MR. Lemierre Syndrome: A Meta-analysis. Int Arch Otorhinolaryngol. 2020;24:e379–85. 32754251 10.1055/s-0039-3402433 PMC7394644

[6] Koton Y Or Z Bisharat N. Septic thrombophlebitis with persistent methicillin-resistant staphylococcus aureus bacteremia and de novo resistance to vancomycin and daptomycin. Infect Dis Rep. 2017;9:7008. 28626538 10.4081/idr.2017.7008 PMC5472341

[7] Gunatilake SS Yapa LG Gallala M. Lemierre’s syndrome secondary to community-acquired methicillin-resistant Staphylococcus aureus infection presenting with cardiac tamponade, a rare disease with a life-threatening presentation: a case report. Int J Emerg Med. 2014;7:39. 25635199 10.1186/s12245-014-0039-y PMC4306077

[8] Loftus MJ Young-Sharma T Wati S. Epidemiology, antimicrobial resistance and outcomes of Staphylococcus aureus bacteraemia in a tertiary hospital in Fiji: a prospective cohort study. Lancet Reg Health West Pac. 2022;22:100438. 35373162 10.1016/j.lanwpc.2022.100438 PMC8969155

[9] Alperstein A Fertig RM Feldman M. Septic thrombophlebitis of the internal jugular vein, a case of Lemierre’s syndrome. Intractable Rare Dis Res. 2017;6:137–40. 28580216 10.5582/irdr.2017.01021 PMC5451747

[10] Abhishek A Sandeep S Tarun P. Lemierre syndrome from a neck abscess due to methicillin-resistant Staphylococcus aureus. Braz J Infect Dis. 2013;17:507–9. 23797007 10.1016/j.bjid.2012.11.010 PMC9428241

[11] Singaporewalla RM Clarke MJ Krishnan PU. Is this a variant of Lemierre’s syndrome? Singapore Med J. 2006;47:1092–5. 17139410

[12] Root RW Barrett TW Abramo TJ. A 10-month-old with Lemierre syndrome complicated by purulent pericarditis. Am J Emerg Med. 2013;31:274.e5–7. 10.1016/j.ajem.2012.05.019 22809766

[13] Jariwala RH Srialluri S Huang MZ. Methicillin-resistant staphylococcus aureus as a cause of lemierre’s syndrome. Pediatr Infect Dis J. 2017;36:429–31. 27977559 10.1097/INF.0000000000001460

[14] Risoud M Mortuaire G Chevalier D. Atypical lemierre syndrome european annals of otorhinolaryngology. Head Neck Dis. 2016;133:123–4. 10.1016/j.anorl.2015.12.001 26718846

[15] Al-Hebshi A Alharbi H Karbouji R. Lemierre syndrome complicating deep neck infection and descending mediastinitis secondary to Methicillin resistant Staphylococcus aureus (MRSA) Infection. Pakistan J Med Sci. 2022;38:1409–12. 10.12669/pjms.38.5.5442 PMC9247774 35799738

[16] Kim BY Yoon DY Kim HC. Thrombophlebitis of the internal jugular vein (Lemierre syndrome): clinical and CT findings. Acta Radiol. 2013;54:622–7. 23528567 10.1177/0284185113481019

[17] Kizhner V Samara G Panesar R. Methicillin-resistant Staphylococcus aureus bacteraemia associated with Lemierre’s syndrome: case report and literature review. J Laryngol Otol. 2013;127:721–3. 23701713 10.1017/S0022215113001035

[18] Nakamichi S Izumikawa K Inoue K. A unique Lemierre syndrome case in an elderly woman with deviation of the tongue. Kansenshogaku Zasshi. 2014;88:704–7. 25672142 10.11150/kansenshogakuzasshi.88.704

[19] Herek PA Lewis T Bailitz JM. An unusual case of lemierre’s syndrome due to methicillin-resistant staphylococcus aureus. J Emerg Med. 2010;39:644–6. 18799283 10.1016/j.jemermed.2007.12.036

[20] Lee W-S Jean S-S Chen F-L. Lemierre’s syndrome: a forgotten and re-emerging infection. J Microbiol Immunol Infect. 2020;53:513–7. 32303484 10.1016/j.jmii.2020.03.027

